# Dynamic changes in Sox2 spatio-temporal expression promote the second cell fate decision through *Fgf4*/*Fgfr2* signaling in preimplantation mouse embryos

**DOI:** 10.1042/BCJ20170418

**Published:** 2018-03-20

**Authors:** Tapan Kumar Mistri, Wibowo Arindrarto, Wei Ping Ng, Choayang Wang, Leng Hiong Lim, Lili Sun, Ian Chambers, Thorsten Wohland, Paul Robson

**Affiliations:** 1 School of Chemical Engineering and Physical Sciences, Lovely Professional University, Phagwara, Punjab 144411, India; 2 Department of Chemistry, National University of Singapore, Singapore; 3 Developmental Cellomics Laboratory, Genome Institute of Singapore, Singapore; 4 MRC Centre for Regenerative Medicine, Institute for Stem Cell Research, School of Biological Sciences, University of Edinburgh, Edinburgh EH16 4UU, U.K.; 5 Department of Biological Sciences, National University of Singapore, Singapore; 6 Centre for Bioimaging Sciences, National University of Singapore, Singapore; 7 The Jackson Laboratory for Genomic Medicine, 10 Discovery Drive, Farmington, CT 06032, U.S.A.

**Keywords:** cooperativity, fluorescence correlation spectroscopy (FCS), Oct4, Sox/Oct motif

## Abstract

Oct4 and Sox2 regulate the expression of target genes such as *Nanog, Fgf4*, and *Utf1*, by binding to their respective regulatory motifs. Their functional cooperation is reflected in their ability to heterodimerize on adjacent *cis* regulatory motifs, the composite Sox/Oct motif. Given that Oct4 and Sox2 regulate many developmental genes, a quantitative analysis of their synergistic action on different Sox/Oct motifs would yield valuable insights into the mechanisms of early embryonic development. In the present study, we measured binding affinities of Oct4 and Sox2 to different Sox/Oct motifs using fluorescence correlation spectroscopy. We found that the synergistic binding interaction is driven mainly by the level of Sox2 in the case of the *Fgf4* Sox/Oct motif. Taking into account *Sox2* expression levels fluctuate more than *Oct4*, our finding provides an explanation on how Sox2 controls the segregation of the epiblast and primitive endoderm populations within the inner cell mass of the developing rodent blastocyst.

## Introduction

The mouse preimplantation embryo is a widely used mammalian model to study cell differentiation. Two of the earliest cell fate decisions in mammalian development take place in the preimplantation embryo. The first decision occurs at the 16–32 cell stage and sets apart the morula into two distinct lineages: the trophoblast, represented by the trophectoderm (TE) and the inner cell mass (ICM). At this stage, the TE is a single layer of epithelial cells enclosing the early blastocyst. The ICM lies at one end of the blastocyst, consisting of a pool of pluripotent cells. Later, after embryonic day 3.5 (E3.5), the ICM is further specified into the primitive endoderm (PE) and epiblast (EPI) lineages [1]. Cells of the PE lineage subsequently differentiate into the extra-embryonic cells responsible for secreting patterning cues and providing nutrition to the developing embryo proper which consists of cells entirely from the EPI lineage.

The EPI is exclusively characterized by its *Nanog and Sox2* expression [2–4], while the PE is specifically characterized by *Gata4*, *Gata6*, and *Sox17* [5–8] and *Oct4* initially persists in both [8]. Prior to the segregation into the PE and the EPI, the ICM shows a mosaic pattern of cells expressing *Nanog* and *Gata6* [9]. The mosaic expression of these markers does not indicate lineage specification as cells expressing the PE markers *Gata6* and *Gata4* can be coaxed into forming the EPI lineage. The cells only become restricted to their definitive lineages at E4.5 [9]. However, studies have also shown that inner cells, which have higher *Nanog* and lower *Gata6* expression, give rise to the EPI while cells with lower levels of *Nanog* and higher levels of *Gata6* give rise to the PE [10,11]. Therefore, it is not clear what role this difference in expression levels of lineage markers plays in the second cell fate decision of preimplantation development. In addition, how this heterogeneity emerges in the first place has also remained elusive. Studies have indicated that the *Fgf4*/*Fgfr2* signaling pathway lies upstream of this differential expression [12–14]. Indeed, *Fgf4* is expressed in the EPI lineage but not in the PE, while *Fgfr2* is expressed in the PE but not in the EPI [15,16]. The segregation of PE from the EPI is also observed to be dependent on FGF/Erk signaling where the entire bipolar ICM can acquire pluripotency if this signal is absent [9,17]. Additionally, a treatment with an Fgf signaling inhibitor causes the otherwise mosaic pattern of the ICM cells to generate exclusively the EPI lineage [13,18]. Recently, it is also reported that p38 family mitogen-activated protein kinases (p38-Mapk14/11) actively participate in the second cell fate determination, especially during early blastocyst maturation for assisting bipolar ICM cells. Interestingly, as like Erk1/2, Fgf-receptor signaling controls the functional activation of p38-Mapk14/11 [19]. Furthermore, both *Fgf4*-null and *Fgfr2*-null embryos are lethal [20,21]. It has been further confirmed that *Fgf4* is required for the segregation of the ICM into the PE and the EPI lineages [13,22,23]. Furthermore, several studies indicate that spatio-temporal differences in inner cell formation contribute to the establishment of the heterogeneity in the ICM [24–26]. Recently, Kang et al. [27] showed that Fgf4 is the central molecule for determining the distinct lineages from ICM cells and Fgf4 imparts its action with the help of Fgfr2 along with Fgfr1 which were shown as critical FGF receptors in establishing the PE lineage. Thus, understanding the molecular determinants that establish this FGF4/FGFR2 signaling axis will shed light on the mechanism that establishes cell fate within the ICM.

In light of the current evidence from mouse preimplantation development, Sox2 emerges as a particularly interesting transcription factor to study. Along with Oct4, it has been found to regulate the expression of other genes important for preimplantation development such as *Nanog*, *Fgf4*, *Utf1*, *Pou5f1*, and *Sox2* itself [28–31]. In the enhancers of these genes, a Sox2-binding motif, CTTTG(A/T)(A/T) [32,33], is found adjacent to an octamer motif, ATGC(A/T)AA(T/A) [34] with a spacer having 0–3 bp in between the two motifs. A recent study also enlightened the importance of an enhancer where it was illustrated that gene activation is highly correlated with the presence of an optimal motif [35]. Furthermore, crystallography studies have shown that the Sox2 and Oct4 DNA-binding domains heterodimerize on this motif [36]. However, unlike *Oct4*, *Sox2* levels show a dynamic pattern in the preimplantation embryo; in particular, zygotic transcription initiates within the inner cells of the morula [13]. Additionally, Sox2 is known to be an activator of *Fgf4* [37] and a repressor of *Fgfr2* [38]. Importantly, Sox2 is required for normal development as Sox2-null embryos fail to develop beyond early post-implantation [39] and is required non-cell-autonomously via FGF4 for the development of the PE [40]. Collectively, these observations indicate that understanding Sox2 dynamics quantitatively is paramount to understanding the molecular mechanism of cell fate decision within the ICM.

We had previously proposed a model based on the dynamics of *Sox2*, *Fgf4*, and *Fgfr2* expression whereby the initiation of Sox2 expression in inner cells of the morula establishes the FGF signaling axis, via the up-regulation of *Fgf4* and the down-regulation of *Fgfr2*, within the ICM [13]. Here, we define the *cis* regulatory logic for this model by measuring the dynamic changes in Sox2 levels through preimplantation development and determining the apparent dissociation constants (a*K*_d_) of Sox2 and Oct4 on their respective *cis* regulatory motifs on target genes of interest. We perform these measurements through the use of fluorescent fusion proteins and fluorescent correlation spectroscopy, a single-molecule sensitive fluorescence-based technique [41,42]. Remarkably, our results reveal that the formation of a stable Sox2–Oct4–DNA complex on the *Fgf4* Sox/Oct motif is more dependent on the level of Sox2 than that on Oct4. Intriguingly, the *Nanog* Sox/Oct motif does not show such a high dependency on the level of Sox2 compared with that of the *Fgf4* Sox/Oct motif. These biochemical measurements lend weight to the argument that Sox2 is indeed the driver of the earliest heterogeneity within the ICM, a heterogeneity that leads to the EPI/PrE cell fate decision.

## Materials and methods

### Electrophoretic mobility shift assay

Electrophoretic mobility shift assay (EMSA) was carried out as described [31,43]. Details of quantitative titration assay are provided in Supplementary Information. Additionally, a fluorescence protein-based EMSA (FP-EMSA) was also performed as described [43,44]. All the oligonucleotides used for EMSA are provided in Supplementary Information.

### Luciferase assay

CHO cells were cultured in Dulbecco's modified Eagle's medium with high glucose (Invitrogen), 10% standard fetal bovine serum (Hyclone), and 1% penicillin/streptomycin and maintained at 37°C with 5% CO_2_. For a 24-well plate, 0.5 µg of EO plasmid, 0.5 µg of MS plasmid, and 0.3 µg of pGL3 NSO plasmid were co-transfected per well using Lipofectamine 2000 (Invitrogen) according to the manufacturer's instructions. *Renilla* luciferase plasmid (0.05 µg; pRL-TK from Promega) was co-transfected as an internal control. Firefly and *Renilla* luciferase activities were measured 24 h post transfection using the Dual Luciferase Kit (Promega) and a Centro LB960 96-well luminometer (Berthold Technologies). Alternatively, F9 embryonal carcinoma (EC) cells were used in the luciferase assay for motif characterization to understand the importance of variable positions in the Sox/Oct motif sequence. Cell culture, transfection, and sample preparation for F9 EC cells were performed as described earlier [31].

### Concentration measurement of fusion protein

The concentration of fusion proteins in unpurified nuclear lysate by fluorescence correlation spectroscopy (FCS) was measured as described [45,46]. See Supplementary Information for the theoretical models used in FCS analysis.

### Data analysis (EMSA and FCS) for a*K*_d_ determination

The apparent dissociation constants, a*K*_d_, were determined as described earlier [46].

### Immunocytochemical staining and Image J-based semi-quantification

Embryos were fixed in 2.5% PFA for 15 min at 37°C, washed with Triton (0.1% in PBS; 5 min), Triton (0.5% in PBS; 20 min), Triton (0.1% in PBS; 5 min), and BSA/Tween (0.1% BSA and 0.01% Tween in PBS; 30 min). After incubation with 1° antibody (Sox2-Y17) in BSA/Tween (60 min), embryos were washed with BSA/Tween (3 × 15 min) and then incubated with 2° antibody (Goat anti-Rabbit IgG) conjugated with Alexa Fluor 488 (Molecular Probes, Carlsbad, CA) for an additional hour. Following BSA/Tween washes (3 × 15 min), embryos were passed through increasing concentrations of mounting solution containing *To-Pro* prior to final mounting. Images were captured with a confocal microscope (LSM 510 META; Zeiss, Thornwood, NJ). All the *Z*-stack images from an individual embryo were grouped into one stack picture based on average fluorescence intensity employing Image J software (NIH). Nuclear staining dye *To-Pro was used as a control for normalizing the fluorescence intensity of* Sox2 targeted antibody tagged with Alexa Fluor 488. It is known that intensity varies with respect to the absolute concentration, and hence, the ratio of fluorescence intensity of Sox2 to that of To-Pro will be the equal to the ratio of concentration of Sox2 to that of To-Pro. The normalized Sox2 concentration ratio, which is an absolute parameter, was compared across the different cell stages of early mouse embryo.

### Time lapse imaging for detection of GFP signal in growing embryos in culture

Two cell-stage embryos, derived by crossing of Sox2-GFP heterozygotic females [47] and males, were cultured in M2 media on the Zeiss microscope (Axio Observer D1, Zeis) stage in appropriate culture conditions (5% CO_2_, 37°C). Fluorescence images were captured in 6 h intervals until the last blastocyst stages. GFP photo-bleaching was minimized by using a low laser power (10 µW) from 488 nm laser. The scale bar was 20 µM.

### Real-time quantitative polymerase chain reaction

All the mouse work was approved by the BRC IACUC (Biopolis). Embryos were derived by crossing of Sox2-GFP heterozygotic females [47] and males and collected at 3.5 dpc in M2 medium. Total RNA was extracted and purified from the whole embryos using a PicoPure RNA isolation kit (Arcturus Bioscience), and cDNA was synthesized with a high capacity cDNA archive kit (Applied Biosystems; ABI). cDNA was first pre-amplified with a pool of 48 inventoried Taqman assays (20×, Applied Biosystems) by denaturing at 95°C for 15 s and annealing and amplification at 60°C for 4 min for 14 cycles. The pre-amplified products were diluted 5-fold and the expressions of the 48 assays were analyzed with 48/48 Dynamic Arrays on a Biomark System (Fluidigm). *C*_t_ values were calculated from the system's software (Biomark Real-time PCR Analysis, Fluidigm). See Supplementary Information for further details of methods and materials part.

## Results

### Sox2 level increases in the ICM with time during preimplantation embryo development

**Figure 1.**
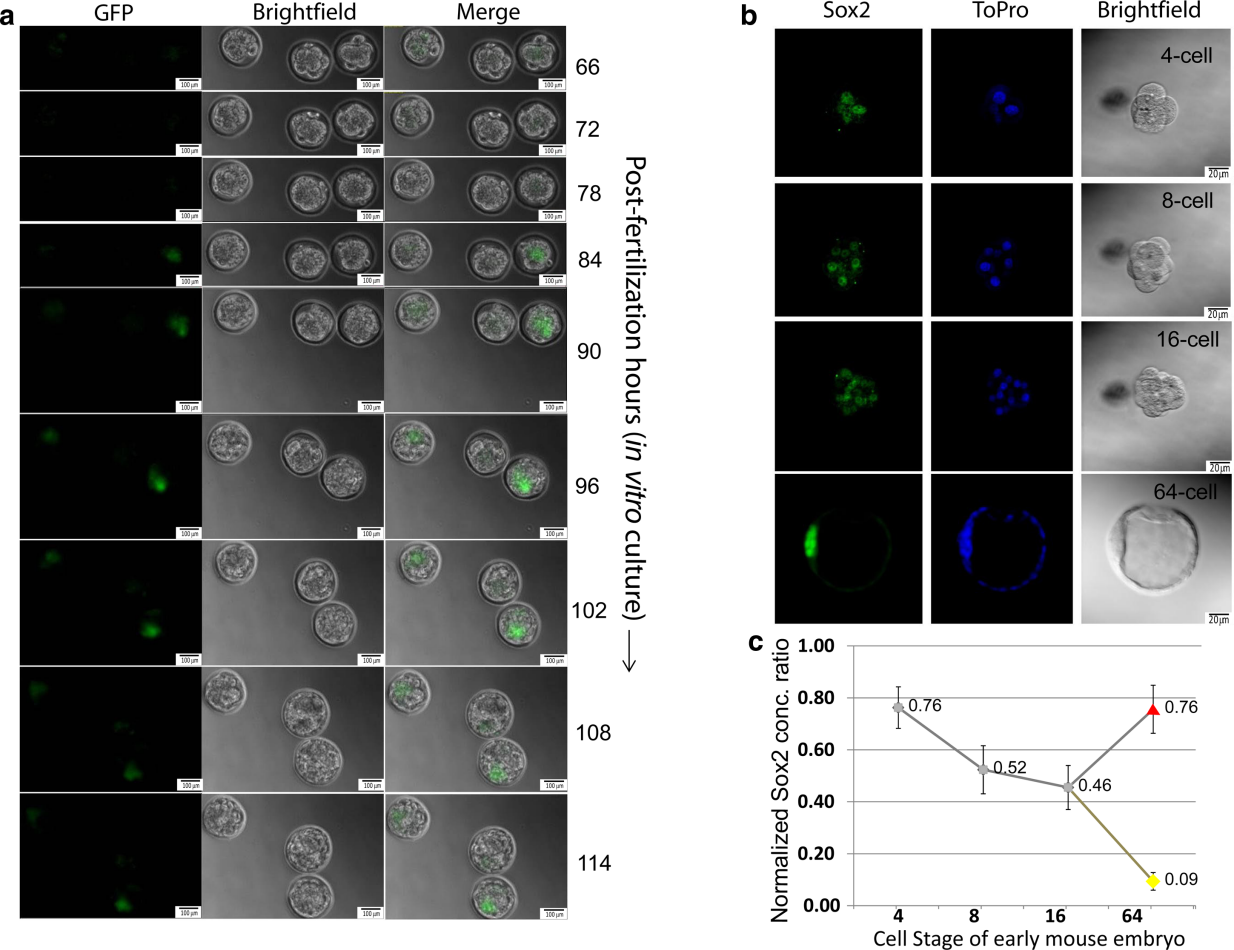
*In vivo* localization and quantification of Sox2 level during mouse preimplantation development. (**a**) Microscopic observation of paternally derived zygotic *Sox2*-GFP expression in *Sox2*-null embryos [13] with the progression of time until 114 h from single cell zygote (guided by an arrow). GFP expression from the *Sox2* locus is observed starting from 66 h at 8-cell stage. The middle embryo is a wild-type control. For each panel, left: GFP, middle: bright field, right: merge. With the progress of experiment, embryos generally move and therefore, panel size was kept bigger in the later stage of the experiment keeping the scale bar same. Scale bars are 20 µm in size in all panels. No. of samples (*S*) = 5, no. of replicates (*R*) = 2. (**b**) Confocal *Z***-**stack images of representative Sox2 and ToPro nuclear stained embryos. *S* = 4, *R* = 2. (**c**) Quantification of the average Sox2 level per cell in 4–16 cell embryos as well as measurements made at the 64-cell stage were from ICM (red triangle) or TE (yellow circle) cells. *S* = 4, *R* = 2, mean ± SEM.

Previously, it has been shown that Sox2 mRNA levels fluctuate more widely than those of Oct4 during preimplantation development [13]. To assess the onset of zygotic Sox2 expression, we measured paternally derived zygotic GFP expression from the *Sox2* locus in *Sox2*-null embryos [47] following progressive cell stages during preimplantation development (Figure 1A). The earliest expression of GFP was found to be within the inner cells of the morula and later restricted to the ICM of the blastocyst. Next, we measured total Sox2 levels by immunostaining (Figure 1B). While Sox2 levels are relatively high at the four-cell-stage levels decrease as development progresses to the morula. Within the blastocyst, Sox2 levels continue to recede in the TE, whereas there is an increase in Sox2 from the morula to ICM (Figure 1C). This Sox2 protein dynamics closely parallels the dynamics of its mRNA level [13]. The impact of this increasing concentration of Sox2 from the morula–ICM transition will be mediated through *cis* regulatory logic.

### Characterization of the Sox/Oct motif sequences

**Figure 2.**
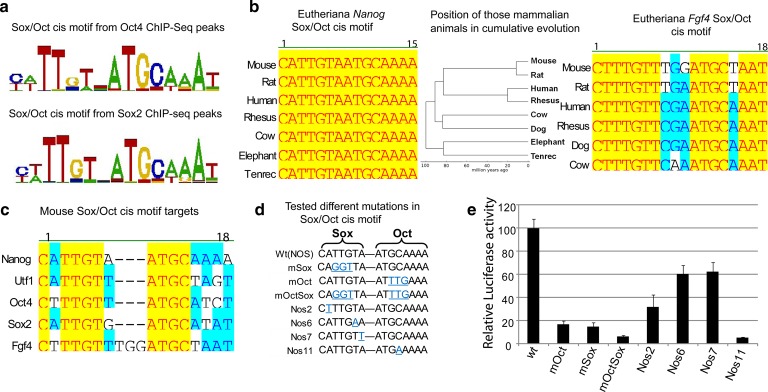
Characterization of Sox/Oct motifs. (**a**) The top *de novo* sequence motifs (based on enrichment) detected by CisFinder [60] in Oct4 and Sox2 ChIP-Seq data from ESCs [49], shown by WebLogo. (**b**) Comparison of *Nanog and Fgf4* Sox/Oct motifs across different eutherian species. A phylogenetic tree illustrated the sequence conservation of those Sox/Oct motifs in cumulative evolution. (**c**) Alignment of the Sox/Oct motifs from the target genes *Nanog*, *Utf1*, *Oct4*, *Sox2*, and *Fgf4*. (**d**) Mutations generated in the Sox/Oct motif in a 400 bp gene fragment from *Nanog* located at −289 to +117 (relative to the *Nanog* transcription start site) [31,61] were subsequently tested. (**e**) Luciferase activity of constructs shown in (**d**) after transfection of F9 embryonal carcinoma cells. *R* = 3, mean ± SD.

Despite the increasing levels of Sox2 in the nascent ICM, some known target genes of Sox2 did not change (e.g. *Nanog*) while others did (e.g. *Fgf4*) [13], and thus, we were next interested in determining if this could be explained through specific variations in *cis* regulatory sequences mediating Sox2 binding. From a global view of Sox2- and Oct4-bound regions in embryonic stem (ES) cells, it is clear that while there is sequence constraint within the Sox/Oct motif, there is allowable variability (Figure 2A). This variability is in contrast with the sequence conservation seen within the particular Sox/Oct motif in both *Nanog* and *Fgf4*, where sequence conservation verges on 100% identity over hundreds of millions of years of cumulative evolution (Figure 2B). Furthermore, we also observed the conservation pattern of different Sox/Oct motifs from the known genes, *Nanog*, *Utf1*, *Oct4*, *Sox2*, *and Fgf4* (Figure 2C). Such sequence conservation strongly argues that there are functional differences between sequences that encompass the allowable Sox/Oct motif.

Through site-directed mutagenesis of the *Nanog* Sox/Oct motif, within the larger context of the *Nanog* 400 bp proximal promoter, we tested the functional consequences of subtle mutations on transcription as measured by luciferase activity generated in transfected F9 teratocarcinoma cells. As previously described [31], ablation of the binding sites for Sox2 or Oct4, and in combination, through 3 bp mutations diminishes luciferase activity to below 20% of the wild-type promoter (Figure 2D,E). When we introduced subtle single-base changes of A to T, T to A, and A to T at positions 2, 6, and 7 of the sox motif, respectively, there was a significant reduction in luciferase activity in all cases from 30 to 60% of wild-type levels (Figure 2D,E). Particularly surprising was the reduction to ∼60% at position 7 of the sox motif as this is the least conserved of all seven positions within the sox motif (Figure 2A,C). Thus, what apparently are subtle changes to the Sox/Oct motif that qualitatively do not prevent binding of Sox2 have profound functional consequences on transcriptional output. We would argue that such functional consequences result in the high level of sequence conservation, through purifying selection, within specific Sox/Oct motifs across eutherian species, particularly around these developmental control genes.

### Characterization of TF-fluorescent fusion proteins

We hypothesized that the differential transcriptional response to increasing concentrations of Sox2 in the nascent ICM seen between *Nanog* and *Fgf4* was a result of differential binding kinetics of Sox2 and Oct4 on the associated *Sox/Oct* motifs of these genes. As no technologies exist to measure protein–DNA-binding kinetics at discrete genomic loci within living cells, we resorted to *in vitro* measurements. Our strategy was to generate quantitative measurements through Förster resonance energy transfer (FRET), FCS, and EMSA on Sox2–Oct4–DNA complexes and thus required the generation of fluorescently tagged proteins.

**Figure 3.**
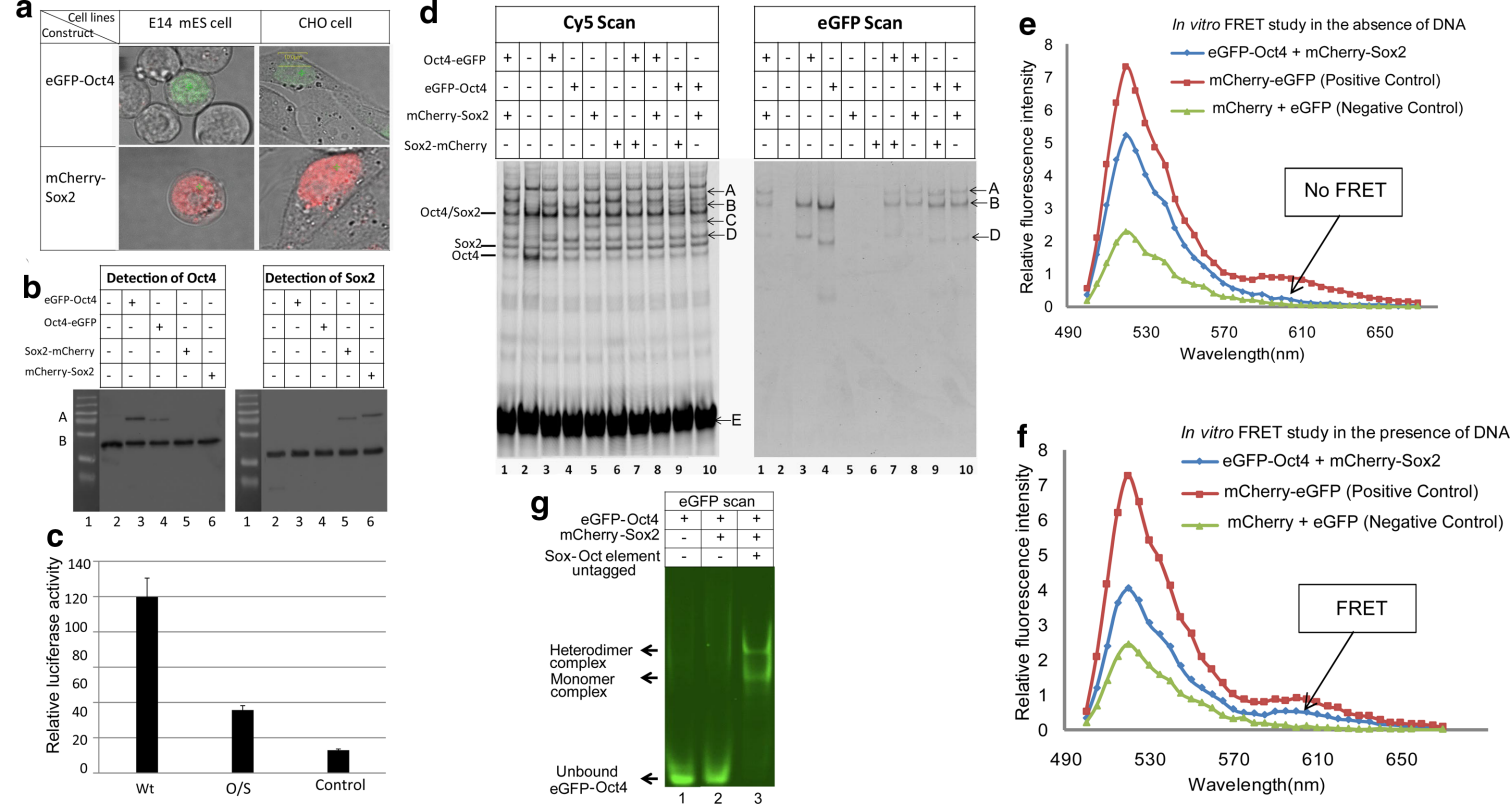
Functional determination of synthetic fusion protein constructs. (**a**) Confocal images of ES cells and CHO cells transfected with pCAG-GFP-Oct4-IN and pCAG-mCherry-Sox2-IN. *R* = 3. (**b**) Immunoblot analysis of exogenous fusion proteins; bands A and B correspond to the fusion and wild-type proteins, respectively. *R* = 3. (**c**) Dual luciferase assays comparing the transcriptional activity of the wild-type *Nanog* Sox/Oct motif (wt) (CATTGTAATGCAAAA), a mutated *Nanog* Sox/Oct motif (O/S) (CAGGTTAATTTGAAA), and a *Nanog* promoter with a deletion form *x*–*y* that removes ONLY the Sox/Oct motif (control). *R* = 2, mean ± SD. (**d**) DNA binding of fusion proteins (GFP scan) compared with wild-type proteins (Cy5 scan) by EMSA from ES cells nuclear lysates. A—heterodimer complex (GFP-Oct4/mCherry-Sox2/NSO motif); B—heterodimer complex (GFP-Oct4/wild-type Sox2/NSO motif); C—monomer complex (mCherry-Sox2/NSO motif); D—monomer complex (GFP-Oct4/NSO motif). *R* = 3. (**e**,**f**) FRET experiments to investigate the interaction of Sox2 and Oct4. As donor we use eGFP-Oct4 and as acceptor mCherry-Sox2. Emission intensity is collected at >500 nm wavelength upon GFP excitation by a 488 nm laser line. FRET analysis was performed on nuclear extracts isolated from transfected CHO cells in the absence (**e**) or the presence (**f**) of DNA containing the Nanog Sox/Oct motif. As indicated in the figure, enhanced emission by mCherry as a donor (see arrows at ∼610 nm) is only evident in the presence of DNA (**f**), implying that Oct4 and Sox2 do only interact via DNA. As a positive control, we used tandem dye eGFP-mCherry; as a negative control, we used co-expression of individual eGFP and mCherry proteins. *R* = 2. (**g**) Complex formation shown by FP-EMSA detecting GFP-Oct4 in the presence or absence of unlabeled DNA and mCherry-Sox2. *R* = 3.

Expression constructs were designed to produce full-length mouse Oct4 and Sox2 fused, via a four amino acid linker (GGSG), with GFP and mCherry, respectively. Initially, we tested functionality of both N-terminal and C-terminal fusions. Transient transfections into mouse ES and CHO cells indicated expression of these transcription factor-fluorescent protein fusion constructs and with nuclear localization (Figure 3A). Western blots, using antibodies against the respective transcription factors, in nuclear lysates from ES cells transfected with these constructs further confirmed the expression of the fusion proteins (Figure 3B). GFP-Oct4 (N-terminal) and mCherry-Sox2 appeared to be expressed at a higher level than their C-terminal-tagged counterparts. Importantly, there were only two types of bands detected, namely for the fusion protein (upper bands close to 75 kDa) and the endogenous protein (lower bands in between 37 and 50 kDa).

We next tested our fusion constructs for their ability to activate transcription in a luciferase promoter assay. CHO cells were used in these experiments as they lack endogenous Oct4 and Sox2 (Supplementary Figure S1). In transient transfection assays, co-transfection of the Oct4 and Sox2 fusion constructs with the wild-type *Nanog* promoter (Wt) resulted in significant luciferase activity above that of the control that lacked the promoter and the mutated promoter (O/S) where mutations applied to the Sox/Oct motif (Figure 3C), indicating that the fusion constructs have the ability to drive transcription from the *Nanog* promoter.

We further sought to determine whether these modified TFs retained the ability to bind DNA similarly to their endogenous counterparts. We tested, by EMSA, nuclear lysates from ES cells transfected with these plasmid constructs for their ability to bind an oligonucleotide containing the *Nanog* Sox/Oct motif. We observed that both endogenous proteins and their fusion counterparts are capable of binding this DNA motif as monomers and heterodimers (Figure 3D). Differentially shifted bands indicate that the fusion proteins can heterodimerise with both their respective endogenous and fusion protein partners. The fusion-containing complexes are readily detectable despite these proteins being expressed at lower levels (Figure 3B) and requiring to compete with their endogenous counterparts in this assay. These results indicate that these fusion proteins are as competent as their wild-type proteins in binding to DNA containing the Sox/Oct motif. Finally, we confirmed the physiological function of the N-terminal fusion proteins, GFP-Oct4 and mCherry-Sox2, for their ability to rescue ES cells in which the corresponding endogenous TF alleles had been deleted [46].

### The Sox2–Oct4 protein–protein interaction requires DNA

Having demonstrated that the Oct4 and Sox2 fusion proteins perform functionally similarly to their endogenous counterparts, we next sought to utilize GFP-Oct4 and mCherry-Sox2 to quantify their combinatorial binding interplay on the Sox/Oct motif. Utilizing FRET we quantitatively investigated the formation of the Sox2 and Oct4 heterodimer complex with the *Nanog* Sox/Oct motif in solution using nuclear extracts from transfected CHO cells. We examined Sox2–Oct4 interactions in the presence and absence of DNA to understand the DNA dependency of Sox2–Oct4 complex formation. No FRET signal was observed from a solution containing GFP-Oct4 and mCherry-Sox2 in the absence of DNA (Figure 3E); however, when DNA containing a *Nanog* Sox/Oct motif was included, a distinct FRET signal was detected (Figure 3F). This observation indicates that the DNA brings GFP-Oct4 and mCherry-Sox2 into close proximity enabling successful energy transfer from GFP to mCherry, through binding to the Sox/Oct motif. In further validation, our FP-EMSA assay also did not detect Sox2–Oct4 interaction unless Sox/Oct DNA was present (Figure 3G) nor are any multimers of GFP-Oct4 detected. These results indicate that heterodimer complexes are only possible in the presence of DNA which is in agreement with our previous work [43,46].

### Determination of apparent dissociation constants (a*K*_d_) by FCS and EMSA

**Figure 4.**
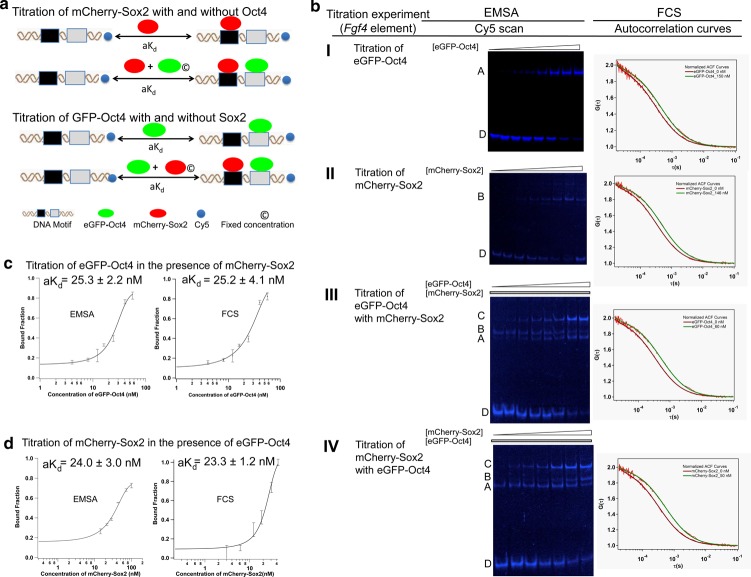
Comparison of FCS and EMSA derived a*K*_d_. (**a**) Conceptual scheme showing the titration of GFP-Oct4 in the absence and presence of mCherry-Sox2 and *vice versa*. (**b**) EMSA scans and autocorrelation curves generated from FCS assays for the *Fgf4* Sox/Oct motif titrating with (I) GFP-Oct4; (II) mCherry-Sox2; (III) GFP-Oct4 in the presence of a fixed mCherry-Sox2 concentration and (IV) mCherry-Sox2 in the presence of a fixed GFP-Oct4 concentration. A: monomer (GFP-Oct4/DNA); B: monomer (mCherry**-**Sox2/DNA); C: heterodimer (GFP-Oct4/mCherry-Sox2/DNA); D: free Cy5-DNA. *R* = 3. (**c**,**d**). Comparison of the bound fraction vs. total protein concentration plots with the *Fgf4* Sox/Oct motif obtained by EMSA (left panel) or FCS (right panel). *R* = 3, mean ± SEM.

Understanding *Fgf4* gene regulation requires the quantitative measurement of mCherry-Sox2 binding to the *Fgf4* Sox/Oct motif compared with that with the *Nanog* and *Utf1* motifs (Supplementary Table S1). For these quantitative studies, we used FCS and EMSA as complementary methods. FCS has been used for many years to measure a*K*_d_ values in lysate, live cells, and zebrafish embryos [46,48]. More recently, we have established FP-EMSA as a complementary technique for quantitative a*K*_d_ measurements using full-length fusion proteins [46]. The titration strategy is shown in Figure 4A. Direct evidence of complex formation on the *Fgf4* Sox/Oct motif under different titration conditions was compared between EMSA generated gel images and FCS generated ACF curves (Figure 4B). In the presence of 72 nM mCherry-Sox2, a titration of GFP-Oct4 to the *Fgf4* Sox/Oct motif yielded a*K*_d_ values of 25.2 ± 4.1 and 25.3 ± 2.2 nM from EMSA and FCS, respectively (Figure 4C). On the other hand, a titration of mChery-Sox2 to the same *Fgf4* Sox/Oct motif in the presence of 40 nM GFP-Oct4 produced a*K*_d_ values of 23.2 ± 1.2 and 24.0 ± 3.0 nM from EMSA and FCS, respectively (Figure 4D). Notably, both FCS and EMSA provided similar values within the margins of standard deviation, strengthening the reliability of our quantitative findings on TF-DNA-binding interactions.

### Influence of sequence variation within the Sox/Oct motif on protein–DNA-binding affinity

Previously, we had observed that sequence changes in the conserved and non-conserved regions of the Sox2 motif had an impact on transcriptional activity (Figure 2E). Such activity may be linked to the degree of Oct4- and Sox2-binding affinities for these DNA motifs (Supplementary Table S1) and consequently affect the formation of a stable heterodimer. The change in the sequence of the Sox2-binding site ‘CATTGTA’ in *Nanog* to ‘CATTGTT’ in *Utf1* revealed a slight decrease in the *Sox2-*binding affinity as the value of a*K*_d_ (decrease in affinity) increased to 44.0 ± 9.8 nM from 31.7 ± 4.6 nM as measured by FCS. The change in the seventh position of the sequence ‘CATTGTA’ in *Nanog* to ‘CATTGTG’ in *Sox2* revealed a slight decrease in the *Sox2-*binding affinity as the a*K*_d_ increased to 66.1 ± 18.2 nM from 44.0 ± 9.8 nM. Additionally, we observed a slight decrease in the *Sox2-*binding affinity when both second and seventh positions were changed to ‘CTTTGTT’ in *Fgf4* from *‘*CATTGTA’ in *Nanog* corresponding to an increase in a*K*_d_ to ∼70 nM (Table 1 and Supplementary Figure S2). Our result demonstrates that the variable positions in the heptamer sequence play an important role in the binding interactions of Sox2 with the Sox/Oct motif, while the conserved positions are key for strong interactions. The variable positions in the Sox2-binding sequence (CtTTGTt) of different Sox/Oct motifs create diversity in DNA-binding affinities of Sox2.

**Table 1:** a*K*_d_ values obtained from titration assays

Sox–Oct element	Apparent dissociation constant, a*K*_d_ (nM)				
	Titration with GFP-Oct4			Titration with mCherry-Sox2	
	No Sox2	Sox2 at 37 nM	Sox2 at 72 nM	No Oct4	Oct4 at 40 nM
Nanog element	28.2 ± 4.9	13.8 ± 3.0 (2.0 ± 0.6)*	11.6 ± 2.4 (2.4 ± 0.7)*	31.7 ± 4.6	13.2 ± 6.5 (2.4 ± 1.2)*
Fgf4 element	42.5 ± 5.5	49.1 ± 5.0 (0.9 ± 0.1)*	25.2 ± 4.1 (1.7 ± 0.4)*	70.2 ± 19.1	23.3 ± 1.2 (3.0 ± 0.8)*
Utf1 element	32.0 ± 5.5	19.8 ± 7.3 (1.6 ± 0.7)*	15.5 ± 6.0 (2.1 ± 0.9)*	44.0 ± 9.8	43.4 ± 1.5 (1.0 ± 0.2)*

The measurement was performed by FCS at constant DNA concentration (5 nM) using unpurified CHO nuclear lysate containing either GFP- Oct4 or mCherry-Sox2. The symbol “*”represents the apparent cooperativity factor.

When we consider the Oct4 motif in terms of its binding affinity, we observed that the first 4 bp, ATGC, in the octamer motif are conserved throughout the five genes. From our luciferase assay (Figure 2C), we know that this conserved region has an important role in Oct4 binding to its motif, but the non-conserved region (fifth to eighth position in the octamer motif) also has an important role in the degree of binding affinity as we have seen from the a*K*_d_ values determined from different Sox/Oct motifs. Comparing between the *Oct4* and the *Sox2* Sox/Oct motifs, we found that the seventh position plays a role in increasing the a*K*_d_ values from 7.7 ± 1.1 to 15.9 ± 1.6 nM. We also looked into the *Utf1* and *Fgf4* motifs and observed that the seventh position plays an important role in increasing the a*K*_d_ value from 32.0 ± 5.5 to 42.5 ± 5.5 nM. The fifth and sixth position displayed a dramatic change when ‘AT’ was replaced by ‘TA’; the value increased to 25 nM, when compared with the *Sox2-* and *Fgf4* Sox2-binding site (Table 1 and Supplementary Figure S2). Therefore, our comparative a*K*_d_ measurements demonstrate the role of variable positions of different Sox/Oct motifs in influencing binding affinity.

Interestingly, we observed that ATGC is highly conserved and thus could be anticipated that ATGC has more influence on Oct4-binding specificity to the octamer sequence of Sox/Oct motifs. Therefore, in addition to FCS affinity measurements, we performed an FP-EMSA with GFP-Oct4 on differently mutated motifs (see Supplementary Information) where we applied mutations in the octamer motif sequence (ATGCAAAA). The results showed that Oct4-binding affinity is more strongly correlated with the first 4 bp of the octamer motif sequence (ATGC) than the last 4 bp (AAAA) (Supplementary Figure S3A and Table S2). We further attempted to understand the effective influence of a single-base pair compared with the collective influence from the conserved base pairs of the octamer sequence (ATGC). In our FP-EMSA experiment, we noted that the sequence AT has a stronger influence than GC (Supplementary Figure S3B and Table S2). These results indicate that the Sox/Oct motif with a base change in the ATGC region would be less potent in binding with Oct4.

### The role of Sox2 concentration on its synergistic interaction with Oct4

Having now established that DNA is necessary for the formation of a stable complex between GFP-Oct4 and mCherry-Sox2, we further investigated the importance of protein concentration on the formation of a stable ternary complex. We measured the *in vitro* a*K*_d_ for mCherry-Sox2 and GFP-Oct4 independently and when in solution together by FCS in two separate titrations (Figure 4A). The main objective was to evaluate whether the ternary complex on the *Fgf4* Sox/Oct motif shows any significant response to the level of mCherry-Sox2. We noted that in the presence of mCherry-Sox2 as a cofactor, GFP-Oct4 showed a higher affinity for the DNA, thus driving exclusive heterodimer formation. Similarly, the presence of GFP-Oct4 as a cofactor aided the binding of Sox2 to these Sox/Oct motifs, thus providing evidence that Oct4 and Sox2 have synergistic effects for *Nanog*, *Fgf4*, and *Utf1* (Table 1). However, there was a significant difference in stable ternary complex recruitment among the *Fgf4*, *Nanog*, and *Utf1* Sox/Oct motifs whereby the *Fgf4* Sox/Oct motif required higher concentrations of Sox2, rather than of Oct4, for the formation of a stable Oct4–Sox2–DNA complex.

The enhanced binding of Oct4 to *Nanog*, *Fgf4*, and *Utf1* depends on the concentration of Sox2 as validated by the increase in the apparent cooperativity factor at the higher Sox2 cofactor concentration (Table 1). The individual titration of the *Fgf4* Sox/Oct motif with Sox2 gave an a*K*_d_ value of 70.2 ± 19.1 nM (Table 1). Owing to the lower binding affinity of Sox2 to the *Fgf4* motif when compared with that of the *Nanog* motif as well as to that of *Utf1*, the influence of Sox2 on Oct4 binding is smaller, giving an apparent cooperativity factor close to 1 at lower Sox2 concentration of 40 nM. At a higher Sox2 concentration of 72 nM, a greater apparent cooperativity factor of 1.7 ± 0.4 was obtained (Table 1). In contrast, the *Nanog* and *Utf1* elements showed a similar synergistic effect at a lower Sox2 concentration of 32 nM. This suggests that a higher Sox2 concentration is essential for increasing the binding affinity of Oct4 for the *Fgf4* Sox/Oct motif. Therefore, from our *in vitro* titration data, we conclude that the *Fgf4* motif needs high levels of Sox2. It has also been reported that *Sox2* and *Fgf4* correlate with each other at the mRNA level during preimplantation development [13]. Consistent with this observation, we too found that the protein levels of Sox2 showed similar trend as seen earlier (Figure 1).

### Comparison of Sox2-binding affinity between *Fgf4* and *Fgfr2 cis* regulatory motifs

**Figure 5.**
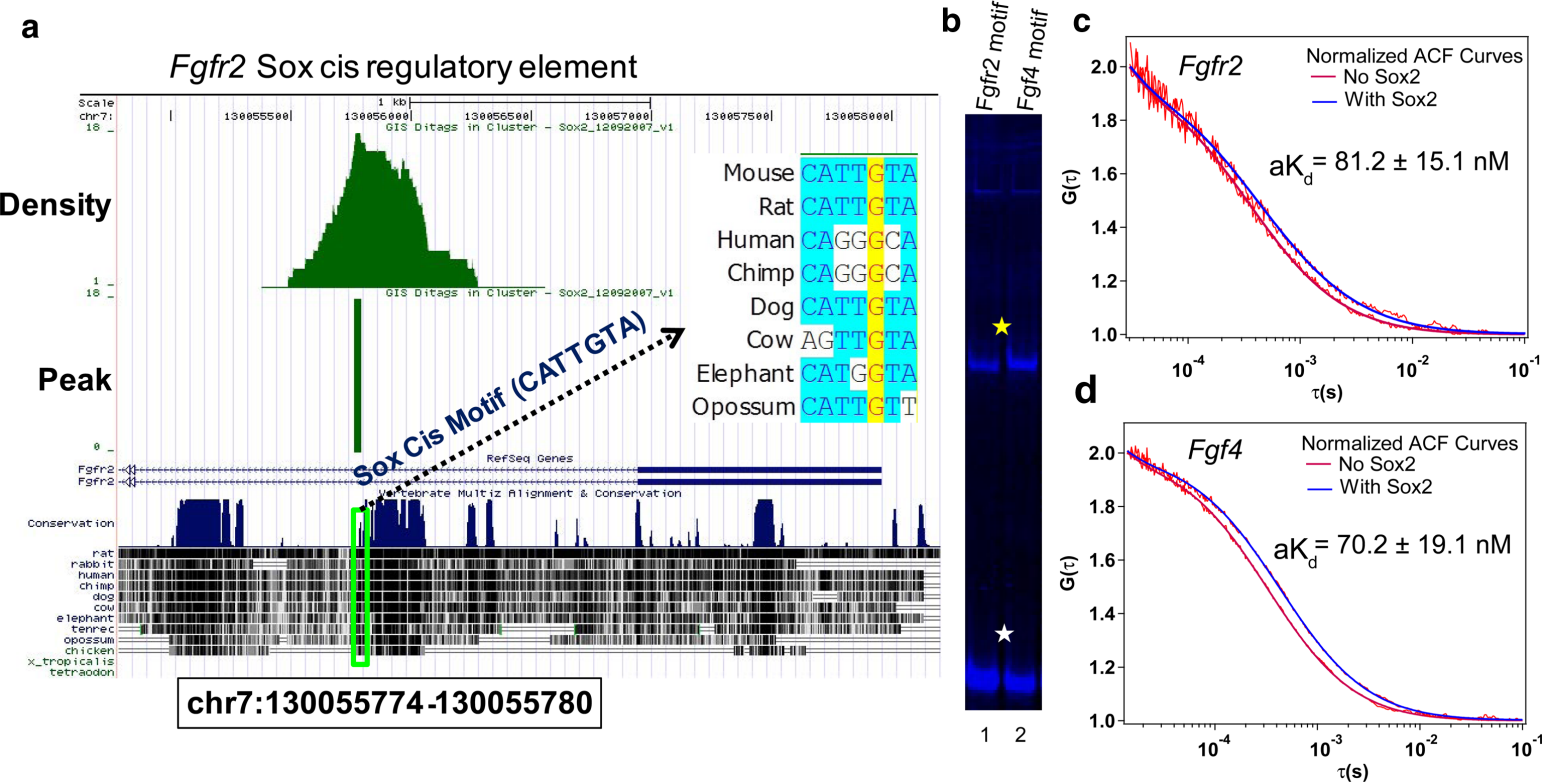
Direct binding of Sox2 protein to *cis* motifs of *Fgf4* and *Fgfr2*. (**a**) Identification of the Sox *cis* regulatory motif near the transcription start site of *Fgfr2* from Sox2 ChIP-Seq data in ES cells [49]. Sequence conservation of the novel motif across different mammalian species is shown. (**b**) EMSA comparison of the binding of Sox2 to the novel Sox *cis* motif and the *Fgf4* Sox/Oct motif; lane 1: *Fgfr2* motif; lane 2: *Fgf4* motif. Yellow and white stars indicate Sox2**–**DNA complex and free DNA, respectively. *R* = 3. (**c**,**d**) Two representative normalized autocorrelation (ACF) curves at no Sox2 as well as at high level of Sox2 shown for *Fgfr2* (**c**) and *Fgf4* (**d**) motifs. The measured a*K*_d_ values from each titration assay measured by FCS technique were compared. *R* = 3, mean ± SEM.

Sox2 is bound to an intronic region of *Fgfr2* in mouse ES cells [49]. We analyzed this data and identified a canonical Sox *cis* motif within this ChIP-seq peak (Figure 5A and Supplementary Figure S4). We analyzed the extent of sequence conservation within this Sox *cis* motif and found it identical in mouse and rats but altered beyond a recognizable Sox motif in human and chimp. This suggests that this is a functional Sox2-binding motif in murine but not in the human genome. Masui et al. [38] had experimentally shown that the removal of Sox2 in mouse ES cells results in *Fgfr2* up-regulation, thus suggesting that Sox2 may be repressing *Fgfr2* expression through this intronic Sox element. We next sought to determine whether Sox2 interacts with this motif in a similar fashion as the *Fgf4* Sox/Oct motif as, in our proposed, model both *Fgf4* and *Fgfr2* expression are sensitive to varying Sox2 concentrations [13]. We therefore performed an EMSA assay using mCherry-Sox2 in CHO nuclear cell lysate with the *Fgfr2* and *Fgf4* motifs. We noted that mCherry-Sox2 formed a stable monomer with both DNA motifs (Figure 5B). We further quantified the binding affinities of Sox2 to these motifs by FCS (Figure 5C,D). Our result showed that both *Fgfr2* (a*K*_d_ value is 81.2 ± 15.1 nM) and *Fgf4* (a*K*_d_ value is 70.2 ± 19.1 nM) require high concentrations of Sox2 for stable complex formation (Figure 5C,D). However, the presence of the Oct4-binding motif in the *Fgf4* Sox/Oct motif favors stable complex formation even at low concentrations of Sox2 (Table 1). This could be the reason for the good correlation between *Fgf4* expression and Sox2 levels during preimplantation development. On the other hand, *Fgfr2* shows minimal expression where the Sox2 level is high such as in the EPI and the opposite happens in the TE lineage. This suggests that Sox2 works as an activator of *Fgf4* and repressor of *Fgfr2.* Therefore, it will be interesting to address further whether the expression of *Fgf4* depends on the level of Sox2 *in vivo*. We next sought to answer this through analysis of Sox2-null embryos.

### Validation of the role of Sox2 on its target genes in Sox2-null embryos by real-time quantitative polymerase chain reaction

**Figure 6.**
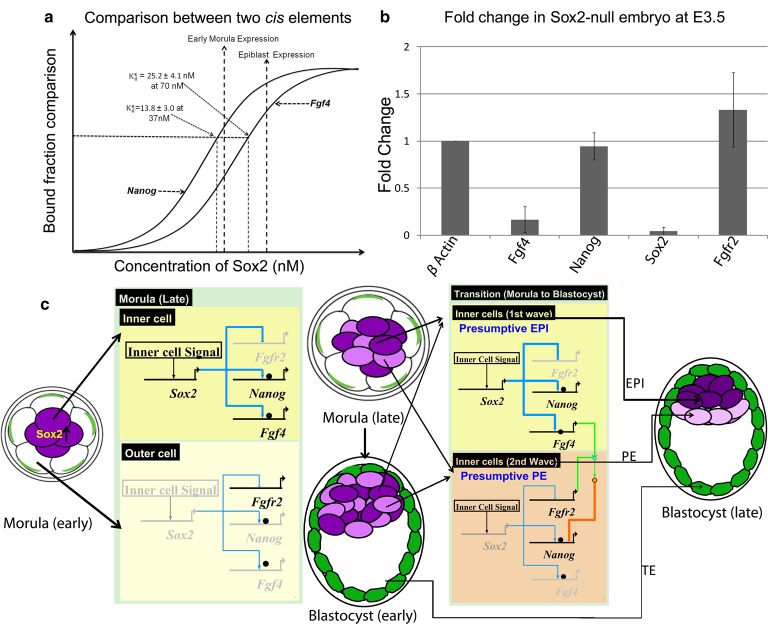
Bridging *in vitro* measurements with *in vivo* Sox2 levels of early embryos. (**a**) Hypothetical diagram showing relation of a*K*_d_ to gene expression with respect to Sox2 concentration is depicted. (**b**) Relative mRNA levels of *Fgf4*, *Nanog*, and *Fgfr2* in *Sox2*-null embryos at E3.5 were compared with that of WT embryos by real-time quantitative polymerase chain reaction. *S* = 4, *R* = 3, mean ± SD. (**c**) Gene regulation model for *Fgf4*, *Nanog*, and *Fgfr2* during the segregation of ICM into PE and EPI by BioTapestry software [62]. The thickness of the blue lines refers to the Sox2 level; the black ball indicates Oct4. Deep and light purple refers to the early inner cells and the late inner cells, respectively. Green shade refers to trophectodermal lineage.

Taken together, our present and published work [13] suggests that Sox2 works as a regulator for both *Fgf4* (positively) and *Fgfr2* (negatively). Zygotic Sox2 expression regulates early embryonic development [39]. To assess the expression of genes confirmed to possess Sox2-interacting regulatory elements, quantitative mRNA analysis was performed in Sox2-null and wild-type embryos, obtained from intercrosses of Sox2^GFP^ heterozygotes [47], after first normalizing expression levels to β-actin. This showed that the Epi marker *Fgf4* was down-regulated significantly in Sox2-null embryos and the PrE marker *Fgfr2* was up-regulated slightly (Figure 6B). We also observed that expression of *Nanog* is not influenced by the absence of Sox2 which further supports our *in vitro* data where we observed that the *Nanog* Sox/Oct motif is capable of making stable ternary complexes at even lower Sox2 concentrations than the *Fgf4* Sox/Oct motif. It should be noted that maternal Sox2 is still present in the early blastocyst [39] and this could be sufficient for the expression of *Nanog* but not for that of *Fgf4* and *Fgfr2* as they require higher Sox2 levels.

## Discussion

Whether the segregation of ICM into PE and EPI is driven by stochastic or deterministic events is currently controversial [9,50]. However, this differentiation event depends on Fgf/Erk signaling which, in turn, depends on communication between inner cells expressing *Fgf4* and inner cells expressing *Fgfr2* [9,13,22,23]. In this study, we provide evidence in support of a model in which temporal alterations in the Sox2 concentration differentially regulate expression of *Fgf4* and *Fgfr2*, thereby driving segregation of ICM into PE and EPI.

To assess key differences in the protein–DNA-binding interactions in the light of what is known about the second differentiation event of mouse embryonic development, we measured the binding kinetics of Oct4 and Sox2 to different Sox/Oct enhancers specific to *Nanog*, *Fgf4*, and *Utf1*. Our *in vitro* protein–DNA-binding analyses indicate that stable protein–DNA complex formation is dependent not only on the DNA sequence specificity but also on the concentration of proteins involved. Interestingly, binding of Sox2 to the *Fgf4* Sox/Oct motif requires a higher concentration of Sox2 (∼2-fold) than is needed for similar complex formation on Sox/Oct motifs from *Nanog* or *Utf1*. Direct binding of Sox2 to the *Fgfr2* and *Fgf4* Sox/Oct motifs and the requirement of a high Sox2 concentration for formation of a stable Sox2/DNA complex further lead us to question whether the expression of *Fgf4/Fgfr2* correlates with the high level of Sox2 *in vivo*. In Sox2-null embryos at E3.5 (and therefore, in the absence of zygotic Sox2), *Fgf4* is down-regulated and *Fgfr2* is up-regulated. These findings argue that *Fgf4/Fgfr2* expression is highly dependent on the Sox2 concentration.

Based on our *in vitro* and *in vivo* results, we propose a model considering *Fgf4* and *Nanog* which adds more clarity to the second cell fate decision during mouse development (Figure 6A). At 37 nM Sox2, the a*K*_d_ value for the *Nanog* Sox/Oct motif is 13.8 ± 3.0 nM with an apparent cooperativity factor of 2.0 ± 0.6 (Table 1), while the value a*K*_d_ for the *Fgf4* Sox/Oct motif is 42.5 ± 5.5 nM with an apparent cooperativity factor of 0.9 ± 0.1 (Table 1). Therefore, at low Sox2 concentration, such as 37 nM, *Nanog* expression may be responsive to Sox2 while *Fgf4* expression may not. This agrees with mRNA expression analysis in Sox2-null embryos (Figure 6B) at E3.5, showing that *Nanog* is expressed but *Fgf4* is not. At 72 nM Sox2, the a*K*_d_ value and apparent cooperativity factor for the *Fgf4* Sox/Oct motif were 25.2 ± 4.1 nM and 1.7 ± 0.4, respectively, whereas the a*K*_d_ and the apparent cooperativity factor for the *Nanog* motif were relatively unchanged. At this elevated Sox2 concentration, both *Nanog* and *Fgf4* may be expressed.

From the above argument, we propose the gene regulation model illustrated (Figure 6C) controlling the segregation of the ICM into the EPI and the PE. As zygotic Sox2 expression is first detectable in the morula, we consider development from the morula to the late blastocyst. The morula, 16-cell-stage embryo, consists of a group of inner cells surrounded by outer cells. In the inner cells, the zygotic expression of Sox2 starts but initially at a concentration not high enough to drive up-regulation of *Fgf4* and down-regulation of *Fgfr2.* In contrast, expression of Sox2 at this stage is absent in the outer cells and the maternal Sox2 protein level is depleting, resulting in down-regulation of *Fgf4* and up-regulation of *Fgfr2*. This scenario becomes more critical for the outer cells at the end of another round of cell division where the Sox2 level decreases further resulting in no expression of *Fgf4* and *Nanog*, but the clear expression of *Fgfr2*. At the end of the 32-cell stage, those outer cells behave like presumptive TE. Prior to the cavitation process, there remains heterogeneity among inner cells, due to a second wave of inner cell formation (derived from earlier outer cells) after cell division of the 16-cell morula. At the end of the 32-cell stage, the early inner cells (deep purple) will already be expressing *Fgf4* and down-regulate *Fgfr2*, whereas the late inner cells (light purple) have high levels of *Fgfr2*. These considerations are consistent with a previous report that also suggested that cells generated in the second wave express higher levels of *Fgfr2* than those from the first wave [51].

After cavitation, the late morula proceeds to the early blastocyst stage where the outer cells are already fated to become TE and the mixed population of early and late inner cells creates the ICM. We assigned to the early inner cells in the ICM as the label of presumptive EPI and to the late inner cells as that of presumptive PE. At this stage of embryo development, the early inner cells possess a high level of *Fgf4*, whereas the late inner cells possess a high level of *Fgfr2*. Therefore, the late inner cells come in direct contact with the signaling output of *Fgf4*-expressing early inner cells, resulting in the down-regulation of many pluripotency genes including *Nanog*, *Klf2*, and *Esrrb* in the late inner cells and up-regulation of PE-specific genes including *Gata6*, *Gata4*, and *Sox17.* Therefore, the late inner cells go on to become the PE lineage. In contrast, the early inner cells maintain the up-regulation of the pluripotency genes and increased zygotic Sox2 levels replenish the depleting level of maternal Sox2 protein. Consequently, they commit to the EPI lineage.

Overall, our working model supports the previously reported ‘time-inside time-outside’ [50–52] or ‘integrated’ cell fate decision model [53], in which ICM cells generated as a result of the 8- to 16-cell transition are biased to form EPI, whereas those generated during the morula to blastocyst (16- to 32-cell) transition are biased to form PE. Our results are consistent with previously reported relative expression levels of *Fgf4* and *Fgfr2* transcripts in inner cells derived from the 8- to 16- and 16- to 32-cell-stage transitions [26] and with a non-cell autonomous role of Sox2 promoting PE development via Fgf4 [40]. In contrast, our findings are not consistent with reports that suggest cell history has little influence on the cell fate decision segregating PE and EPI [18,22]. Notably, however, while Ohnishi et al. [22] argue that this cell fate decision is stochastic, they do find bimodal expression of *Fgf4* within the earliest ICM stage they analyzed. We argue that this bimodal expression of *Fgf4* is a result of inner cell-specific initiation of zygotic Sox2 expression combined with the sensitivity of the Sox/Oct *cis* regulatory motif within *Fgf4* to Sox2 levels. This second cell fate decision is linked to the first cell fate decision via hippo signaling as expression of zygotic Sox2 in inner cells requires active hippo signaling, which in turn is controlled by the relative positioning of cells within the morula [40,54–58]. It is also worth mentioning the Sox2-bound, repressively acting *cis* motif we identify in *Fgfr2* is conserved in rodents, but unrecognizable in humans — this provides a feasible explanation for the observation that cell fate decision within the human ICM is independent of Fgf signaling [59].

In summary, the *in vitro* protein–DNA-binding data and *in vivo* analysis of Sox2 levels controlling the expression of *Fgf4* and *Fgfr2* allows us to argue that Sox2 increasing levels in the inner cells could be a determinant for the segregation of the ICM into the PE and the EPI cell lineages.

## Abbreviations

E3.5: embryonic day 3.5
EC: embryonal carcinoma
EMSA: electrophoretic mobility shift assay
EPI: epiblast
ES: embryonic stem
FCS: fluorescence correlation spectroscopy
FP-EMSA: fluorescence protein-based EMSA
FRET: Förster resonance energy transfer
ICM: inner cell mass
PE: primitive endoderm
TE: trophectoderm
