# Supplementary material for: Dynamic changes in Sox2 spatio-temporal expression promote the second cell fate decision through Fgf4/Fgfr2 signaling in preimplantation mouse embryos
Source: Biochem J. 2018 Mar 20;475(6):1075–89. doi: 10.1042/BCJ20170418 (PMC5896025; doi:10.1042/BCJ20170418)
Supplement: Supplementary Tables and Figures [file BCJ-475-1075-s1.pdf]

Dynamic changes in Sox2 spatio-temporal expression promote the second cell fate decision through Fgf4/Fgfr2 signalling in preimplantation mouse embryos

Tapan Kumar Mistri et al. 2018

Table of contents:

Content	Page(s)
FLUORESCENCE CORRELATION SPECTROSCOPY (THEORY)	1-2
EXTRA MATERIALS AND METHODS	2-5
FUSION PROTEIN DESIGN	5-6
BINDING ASSAY FOR TITRATION	6-6
OLIGONUCLEOTIDES FOR DIFFERENT EMSA ASSAYS	6-7
SUPPLEMENTARY FIGURES S1, S2, S3 & S4	7-11

FLUORESCENCE CORRELATION SPECTROSCOPY (THEORY)

FCS is a single molecule sensitive technique that provides quantitative information on molecular diffusion and concentration of fluorescent molecules within the sample. Fluorescence tagged molecules which diffuse through the confocal volume by Brownian motion generate fluorescence fluctuations. This fluctuation is converted into an autocorrelation curve by a normalized autocorrelation function (ACF):

$$G(\tau) = \frac{\langle F(t)F(t+\tau)\rangle}{\langle F(t)\rangle^2}$$

Equation (1)

In a binary binding experiment, where a fraction of the labelled molecules is unbound, (free) thereby exhibiting faster diffusion characteristics than a bound molecule in a binding complex, the experimental ACF is then fitted by a theoretical ACF describing two species with different diffusion times τ_D undergoing 3D free diffusion [1].

$$G(\tau) = \frac{1}{N}\left((1-y)\left(1+\frac{\tau}{\tau_{free}}\right)^{-1}\left[1+\left(\frac{\omega_0}{z_0}\right)^2\frac{\tau}{\tau_{free}}\right]^{-1/2} + y\left(1+\frac{\tau}{\tau_{bound}}\right)^{-1}\left[1+\left(\frac{\omega_0}{z_0}\right)^2\frac{\tau}{\tau_{bound}}\right]^{-1/2}\right) + G(\infty)$$

Equation (2)

Where N is the total number of molecules undergoing the different diffusion in the observation volume; ω_0 and z_0 are the radial and axial distances where the excitation intensity reaches $1/e^2$ of its value from the centre of the observation volume; τ_D is the diffusion time of the fluorophore through the observation volume; $G(\infty)$ is the convergence value of the autocorrelation curves for long times with the expected value of 1; *y* is the fraction of the bound species undergoing diffusion time of τ_{Di}.

The diffusion time τ_D reflects the size of the fluorescent tagged molecule, and in the case of binding, the size of the complex. It is related to the diffusion coefficient *D* by

$$\tau_D = \frac{{\omega_0}^2}{4D}$$

Equation (3)

The effective observation volume V_{eff} can be determined by using a fluorescent dye of known D, by first obtaining ω_0 followed by z_0 (from Equation (2) and (3)):

$$V_{eff} = \pi^{\frac{3}{2}} \omega_0^{\ 2} \omega_z$$

Equation (4)

Lastly, the absolute concentration (in molar unit (M)) can be obtained by:

$$C = \frac{N}{N_A V_{eff}}$$

Equation (5)

N is the total number of fluorophores in the effective volume, V_{eff}, N_A is the Avogadro constant which is 6.022×10^{23}.

MATERIALS AND METHODS

Fusion Protein Design and Expression Vector Construction – Mouse Oct4 and Sox2 proteins were fused at their amino termini with the fluorescent proteins GFP and mCherry respectively to generate GFP-Oct4 and mCherry-Sox2 fusion proteins. Two rounds of PCR using the Expand High Fidelity PCR System (Roche) were performed to obtain the fusion construct**.** An initial round was performed for each protein separately using an outer primer to append the sites for NotI or SacI and an inner primer to append the linker sequence, encoding Gly-Gly-Ser-Gly, and a portion of nucleotide sequence encoding a part of the partner protein. A similar procedure was employed to make the Oct4-GFP and the Sox2-mCherry C-terminal fusions. The primers used for the initial PCR and Fusion PCR are shown below;

**Plasmid (pCAG backbone)**	**Primers**	**5'-3'**
pCAG GFP-Oct4 (N terminal fusion)	1F GFP	AATTGAGCTCTATGGCCACAACCATGGTGAGCAAGGGCGAGGAGC
	1R GFP	GAAGCCAGGTGTCCAGCCATGCCGCTGCCGCCCTTGTACAGCTCGTCCATGCC
	2F Oct4	GGCATGGACGAGCTGTACAAGGGCGGCAGCGGCATGGCTGGACACCTGGCTTC
	2R Oct4	AGTCGCGGCCGCTTCAGTTTGAATGCATGGGAG
pCAG Oct4-GFP (C terminal fusion)	1F Oct4	AATTGAGCTCCCGTCCCTAGGTGAGCCG
	1R Oct4	CCTCGCCCTTGCTCACCATGCCGCTGCCGCCGTTTGAATGCATGGGAGAGC

	2F GFP	GCTCTCCCATGCATTCAAACGGCGGCAGCGGCAT GGTGAGCAAGGGCGAGG
	2R GFP	AGTCGCGGCCGCTTTACTTGTACAGCTCGTCC
pCAG mCherry-Sox2 (N terminal fusion)	1F mCherry	AATTGAGCTCTATGGCCACAACCATGGTGAGC
	1R mCherry	GCTTCAGCTCCGTCTCCATCATGCCGCTGCCGCCC TTGTACAGCTCGTCCATGC
	2F Sox2	GCATGGACGAGCTGTACAAGGGCGGCAGCGGCAT GATGGAGACGGAGCTGAAGC
	2R Sox2	AGTCGCGGCCGCTTCACATGTGCGACAGGGGCAG
pCAG Sox2-mCherry (C terminal fusion)	1F Sox2	AATTGAGCTCCCAGCGCCCGCATGTATAACATG
	1R Sox2	CCTCCTCGCCCTTGCTCACCATGCCGCTGCCGCCC ATGTGCGACAGGGGCAGTG
	2F mCherry	CACTGCCCCTGTCGCACATGGGCGGCAGCGGCAT GGTGAGCAAGGGCGAGGAGG
	2R mCherry	AGTCGCGGCCGCTTTACTTGTACAGCTCGTCCATG C

Five fusion proteins were designed as follows:

1. Oct4 fused in its C-terminal with GFP (pCAG-Oct4-GFP-IN)
2. Oct4 fused in its N-terminal with GFP (pCAG-GFP-Oct4-IN)
3. Sox2 fused in its C-terminal with mCherry (pCAG-Sox2-mCherry-IN)
4. Sox2 fused in its N-terminal with mCherry (pCAG-mCherry-Sox2-IN)
5. mCherry fused in its C-terminal with GFP (pCAG-mCherry-GFP-IN), for FRET as Positive control

All fusion proteins are cloned into a pCAG vector to drive its expression in mouse embryonic stem cells.

These below three vectors were constructed without fusion PCR for FRET experiment.

1. GFP (pCAG-GFP-IN), for GFP
2. mCherry (pCAG-mCherry-IN), for mCherry
3. GFP-IRES-mCherry (pCAG-GFP-IRES-mCherry-IN), where IRES (Internal Ribosome Entry Site) allows GFP and mCherry to be expressed separately.

[**NOTE**: In all cases, IN stands for IRES-Neomycin cassette attached next to the protein of interest. In addition, GFP protein, used in this study, contains a mutation (alanine to lysine at amino acid 206) [2, 3] for reducing its tendency to form homodimers.]

Cell culture and transfection – ES cells were grown on a layer of mouse embryonic fibroblast in Dulbecco's modified Eagle's medium (DMEM, GIBCO-19600), 20% ES standard fetal bovine serum (FBS), 1x non-essential amino acids, 2 mM L-glutamine, 1X penicillin/streptomycin 0.1 mM 2-mercaptoethanol, and an aliquot of recombinant LIF conditioned medium. CHO-K1 cells (ATCC # CRL-61) were grown in Dulbecco's modified Eagle's medium (DMEM, GIBCO-19600), 10 % fetal bovine serum (GIBCO), 1x non-essential amino acids, 2 mM L-glutamine, 1X penicillin/streptomycin. The incubator conditions were set to 37 ºC, 5 % CO_2 and 95 % humidity. Transfections of required plasmids were performed using Lipofectamine 2000 (Invitrogen) according to the manufacturer's instructions. 24 µg of plasmid was transfected into CHO cells on 59 cm^2 plates. The cells were collected after 24 hours to prepare nuclear cell lysates.

Nuclear lysate preparation Nuclear extracts were prepared as described [4, 5].

Western blotting-Western blotting was performed using nuclear cell lysate (E14 mES and CHO cells line). DNA plasmids encoding the required TFs were transfected using Lipofectamine 2000 (Invitrogen) according to the manufacturer's instructions. Cells were harvested after 24 hrs and nuclear lysates prepared. The blot was run following a standard protocol. Sox2 and Oct4 fusion proteins were detected using the following antibodies: for Oct4 Oct4-N19 primary (obtained from goat, Santa Cruz Biotechnology) (0.5 µl in 10 ml 5% blocker solution), for Sox2 Sox2-Y17 primary (obtained from goat, Santa Cruz Biotechnology) (2 µl in 10 ml %5 blocker solution). HPR conjugated rabbit anti-goat antibody (Santa Cruz Biotechnology) was used as secondary antibody in both cases. Signal development was done by incubating in super signal® west pico chemo luminescent substrate mixture [stable peroxide solution and luminal enhancer solution] (Pierce cat. # 34077) for 5 minutes. The final image was obtained using VersaDoc instrument (BioRad).

BINDING ASSAY FOR TITRATION

BINDING REACTIONS FOR TITRATION USED IN FCS AND EMSA									
**In the absence of co-factor**	Volume (μl)								
Protein (EO or MS)	0.0	0.5	1.0	1.5	2.0	3.0	5.0	7.0	9.0
BBC (make up to 9ul)	9.0	8.5	8.0	7.5	7.0	6.0	4.0	2.0	0.0
Cy5-NSO (150nM)	0.5	0.5	0.5	0.5	0.5	0.5	0.5	0.5	0.5
BB (80% glycerol)	1.0	1.0	1.0	1.0	1.0	1.0	1.0	1.0	1.0
dGdC (2ug/ul)	1.5	1.5	1.5	1.5	1.5	1.5	1.5	1.5	1.5
Total volume	12.0	12.0	12.0	12.0	12.0	12.0	12.0	12.0	12.0

**In the presence of co-factor**	Volume (μl)								
Protein (EO or MS)	0.0	0.5	1.0	1.5	2.0	3.0	5.0	7.0	8.0
BBC (make up to 9ul)	7.0	6.5	6.0	5.5	5.0	4.0	2.0	1.0	0.0
Cofactor (MS or EO)	5.0	5.0	5.0	5.0	5.0	5.0	5.0	5.0	5.0
Cy5-NSO (150nM)	0.5	0.5	0.5	0.5	0.5	0.5	0.5	0.5	0.5
BB (80% glycerol)	1.0	1.0	1.0	1.0	1.0	1.0	1.0	1.0	1.0
dGdC (2ug/ul)	1.5	1.5	1.5	1.5	1.5	1.5	1.5	1.5	1.5
Total volume	15.0	15.0	15.0	15.0	15.0	15.0	15.0	15.0	15.0

EO: GFP-Oct4, MS: mCherry-Sox2, BBC: Binding Buffer C, BB: Binding Buffer.

OLIGO SEQUENCES FOR QUANTITATIVE ASSAYS

Name (motif)	Sequence	Figure
Nanog -SO	TCCACCATGGA**CATTGTAATGCAAAA**GAAGCTGTAAG	Fig. 3, Fig. S3, Table-1
Utf1-SO	GGATGAGCCGT**CATTGTTATGCTAGT**GAAGTGCCGGC	Fig. S3, Table-1
Fgf4-SO	TAGAAAACT**CTTTGTTTGGATGCTAAT**GGGATACTTA	Fig. 4, Fig. 5, Fig. S3, Table-1
Oct4-SO	CTATCATGCAC**CTTTGTTATGCATCT**GCCGTCTGCCC	Fig. S3
Sox2-SO	CTCGGGCAGC**CATTGTGATGCATAT**AGGATTATTCA	Fig. S3
Fgfr2-S	GCCACCCCACTTCATTGTAGCATTTCTTTCATTTGTC	Fig. 5

Supplementary Table 1: Oligonucleotides used for titration assays. DNAs used are shown 5'-3'. In red is the Sox2 binding site and in green is that of. All the motifs are labeled with Cy5.

OLIGO SEQUENCES FOR FP-EMSA

Name (motif)	Sequence	Figure
NSO	GGACATTGTAATGCAAAAGAA	Fig. S3a-b
NSO_mut	GGACAaataAgcctAAAAGAA	
NSO _mO5-8C	GGACATTGTAATGCccccGAA	Fig. S3a
NSO _mO5-8T	GGACATTGTAATGCttttGAA	
NSO _mO5-8G	GGACATTGTAATGCggggGAA	
NSO _mO1-4A	GGACATTGTAAaaaAAAAGAA	
NSO _mO1-4G	GGACATTGTAggGgAAAAGAA	
NSO _mO1-4C	GGACATTGTAcccCAAAAGAA	
NSO _mO1-4T	GGACATTGTAtTttAAAAGAA	
NSO_O1	CATGGACATTGTAcTGCAAAAGAAGCT	Fig. S3b
NSO_O2	CATGGACATTGTAAaGCAAAAGAAGCT	
NSO_O3	CATGGACATTGTAATaCAAAAGAAGCT	
NSO_O4	CATGGACATTGTAATGgAAAAGAAGCT	
NSO_O1-2	CATGGACATTGTAcaGCAAAAGAAGCT	
NSO_O3-4	CATGGACATTGTAATagAAAAGAAGCT	
NSO_O1-4	CATGGACATTGTAcaagAAAAGAAGCT	

Supplementary Table 2: Oligonucleotides for testing sequence specificity of Oct4 binding to *Nanog* Sox/Oct (NSO) motif. DNAs used are shown 5'-3'. In red is the Sox2 binding site and in green is that of Oct4. Mutations are marked by lower case (substitutions). All the motifs here are unlabeled (unless otherwise mentioned in the Figure legend).

SUPPLEMENTARY FIGURES

SUPPLEMENTARY FIGURE 1.

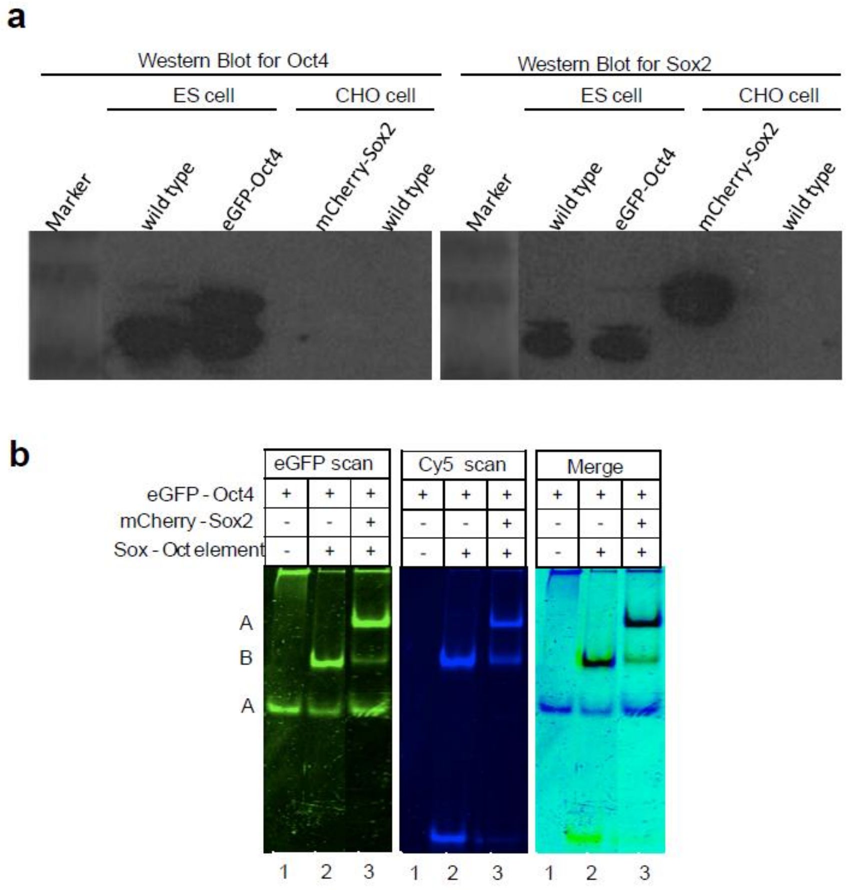

SUPPLEMENTARY FIGURE 1. **CHO cell line is ideal for fusion protein generation.** (**a**) Western blot results for Oct4 (left) and Sox2 (right) in ES or CHO nuclear cell lysates. R = 3. (**b**) Complex formation of GFP-Oct4 with the *Nanog* Sox/Oct motif in the presence or absence of mCherry-Sox2 by FP-EMSA. R = 3.

SUPPLEMENTARY FIGURE 2.

	Sox2 binding site							aK_d (nM)
	1	2	3	4	5	6	7	
Nanog	C	A	T	T	G	T	A	31.7 ± 4.6
Utf1	C	A	T	T	G	T	T	44.0 ± 9.8
Oct4	C	T	T	T	G	T	T	72.3 ± 9.9
Sox2	C	A	T	T	G	T	G	66.1 ± 18.2
Fgf4	C	T	T	T	G	T	T	70.2 ± 19.1

	Oct4 binding site								aK_d (nM)
	1	2	3	4	5	6	7	8	
Nanog	A	T	G	C	A	A	A	A	28.2 ± 4.9
Utf1	A	T	G	C	T	A	G	T	32.0 ± 5.5
Oct4	A	T	G	C	A	T	C	T	7.7 ± 1.1
Sox2	A	T	G	C	A	T	A	T	15.9 ± 1.6
Fgf4	A	T	G	C	T	A	A	T	42.5 ± 5.5

SUPPLEMENTARY FIGURE 2. **aK_d of Sox/Oct motifs measured for either Oct4 or Sox2**. The indicated Sox/Oct motifs were compared to show the Sox2 binding site sequence (left) and the Oct4 binding site sequence (right) as well as aK_d values measured for either Sox2 or Oct4 alone by FCS. R = 3, mean ± SEM.

SUPPLEMENTARY FIGURE 3.

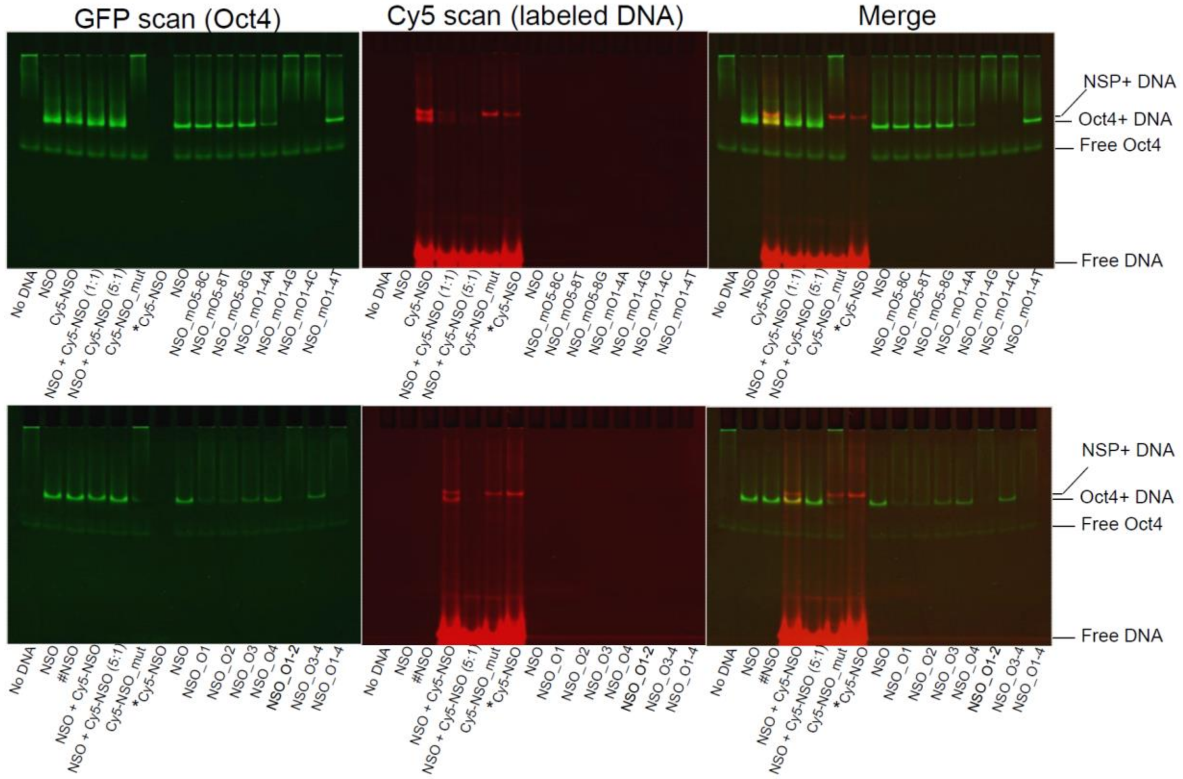

SUPPLEMENTARY FIGURE 3. **Importance of sequence specificity in Oct4-DNA binding on Nanog Sox/Oct motif.** (**a**) The binding affinity of Oct4 to the *Nanog* Sox/Oct motif or to mutations within the octamer (ATGCAAAA) sequence is compared. Controls are shown in lanes 1 to 8 and mutation experiments in lanes 9 to 15. GFP-Oct4 was added in all lanes except lane 7 (untransfected CHO nuclear lysate). (**b**) The binding affinities of Oct4 to the *Nanog* Sox/Oct *cis* motif or to different mutations within the octamer (ATGC) sequence were compared. Controls were shown in lanes 1 to 8 and mutation experiments in lanes 9 to 15. GFP-Oct4 was added in all lanes except lane 7 (untransfected CHO nuclear lysate). All oligonucleotide sequences are in the **supplementary information**. R = 2

SUPPLEMENTARY FIGURE 4.

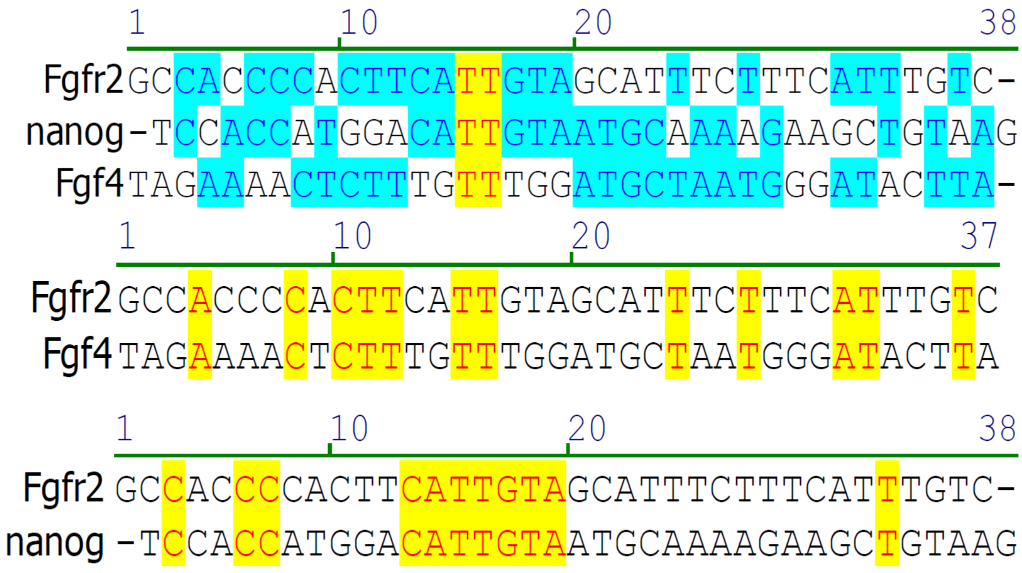

SUPPLEMENTARY FIGURE 4. **Comparison of natural DNA sequences among *Fgfr2*, *Fgf4*, and *Nanog* motifs**. 37 bps long DNA elements containing either of *Fgfr2*, *Fgf4*, or *Nanog* motif were compared for all three oligos (top), *Fgfr2* and *Fgf4* (middle) and *Fgfr2* and *Nanog* (bottom). Yellow colour represents base pair similarity among variants and blue colour represents base pair similarity for less than 3 variants.

REFERENCE

1 Kinjo, M. and Rigler, R. (1995) Ultrasensitive hybridization analysis using fluorescence correlation spectroscopy. Nucleic acids research. **23**, 1795

2 Shaner, N. C., Steinbach, P. A. and Tsien, R. Y. (2005) A guide to choosing fluorescent proteins. Nat Methods. **2**, 905-909

3 Zacharias, D. A., Violin, J. D., Newton, A. C. and Tsien, R. Y. (2002) Partitioning of lipid-modified monomeric GFPs into membrane microdomains of live cells. Science. **296**, 913

4 Andrews, N. C. and Faller, D. V. (1991) A rapid micropreparation technique for extraction of DNA-binding proteins from limiting numbers of mammalian cells. Nucleic acids research. **19**, 2499

5 Mistri, T. K., Devasia, A. G., Chu, L. T., Ng, W. P., Halbritter, F., Colby, D., Martynoga, B., Tomlinson, S. R., Chambers, I. and Robson, P. (2015) Selective influence of Sox2 on POU transcription factor binding in embryonic and neural stem cells. EMBO reports. **16**, 1177-1191
